# Analysis of the functional compatibility of SIV capsid sequences in the context of the FIV gag precursor

**DOI:** 10.1371/journal.pone.0177297

**Published:** 2017-05-05

**Authors:** César A. Ovejero, José L. Affranchino, Silvia A. González

**Affiliations:** Laboratorio de Virología, Consejo Nacional de Investigaciones Científicas y Técnicas (CONICET)-Universidad de Belgrano (UB), Buenos Aires, Argentina; University of Alabama at Birmingham, UNITED STATES

## Abstract

The formation of immature lentiviral particles is dependent on the multimerization of the Gag polyprotein at the plasma membrane of the infected cells. One key player in the virus assembly process is the capsid (CA) domain of Gag, which establishes the protein-protein interactions that give rise to the hexagonal lattice of Gag molecules in the immature virion. To gain a better understanding of the functional equivalence between the CA proteins of simian and feline immunodeficiency viruses (SIV and FIV, respectively), we generated a series of chimeric FIV Gag proteins in which the CA-coding region was partially or totally replaced by its SIV counterpart. All the FIV Gag chimeras were found to be assembly-defective; however, all of them are able to interact with wild-type SIV Gag and be recruited into extracellular virus-like particles, regardless of the SIV CA sequences present in the chimeric FIV Gag. The results presented here markedly contrast with our previous findings showing that chimeric SIVs carrying FIV CA-derived sequences are assembly-competent. Overall, our data support the notion that although the SIV and FIV CA proteins share 51% amino acid sequence similarity and exhibit a similar organization, i.e., an N-terminal domain joined by a flexible linker to a C-terminal domain, their functional exchange between these different lentiviruses is strictly dependent on the context of the recipient Gag precursor.

## Introduction

Lentiviral assembly at the plasma membrane of the infected cells results from the multimerization of the Gag polyprotein into particles that then bud into the extracellular medium (reviewed in refs. [1,2]). The Gag precursors of simian and feline immunodeficiency viruses (SIV and FIV, respectively) consist of the three functionally conserved domains among retroviruses, matrix (MA), capsid (CA), and nucleocapsid (NC), as well as a C-terminal domain (p6 in SIV; p2 in FIV) [3]. These domains are linked by spacer peptides located between CA and NC (SP1 in SIV; p1 in FIV) and between NC and p6 (SP2 only in SIV). The modular nature of Gag enables this viral protein to play multiple roles during the assembly and budding of viral particles: the N-terminal MA mediates the targeting and association of Gag with the plasma membrane [4–9] and is also involved in the packaging of the SIV and human immunodeficiency virus type 1 (HIV-1) envelope (Env) glycoproteins into virions [3,10–13]; the central CA-SP1 domain establishes the Gag-Gag interactions that result in the hexagonal lattice of the spherical immature virion [14–20]; and the NC domain, through its two zinc finger motifs, selectively encapsidates the viral genomic RNA, which also provides a nucleation scaffold for Gag assembly [21–27]. In addition, both SIV and FIV Gag C-terminal domains interact with components of the endosomal-sorting complexes required for transport (ESCRT), thereby promoting the release of virus particles from the plasma membrane of infected cells [26,28–30]. It is worth mentioning that in the case of HIV-1, virion budding has been shown to be dependent on the functional cooperation between p6 and NC, since the latter appears to also bind to ESCRT components [31–33]. Concomitantly with immature virion budding, the viral protease-mediated cleavage of Gag into its functional domains leads to the formation of the mature infectious particle in which the CA protein rearranges into the core that encloses the NC-genomic RNA complex [1,2]. Therefore, lentiviral assembly is tightly dependent on the CA. Indeed, as a domain of the Gag precursor, the CA mediates the formation of the Gag lattice in the immature particle, whereas as an independent structural protein, it assembles into the fullerene core structure that distinguishes the mature infectious virion.

However, the roles of the CA are not limited to virion assembly: the mature CA is also a key player in virion uncoating and nuclear import of the preintegration complex [34–37]. Moreover, it has recently been shown that the HIV-1 CA lattice creates positively charged pores that allow the recruitment of nucleotides into the capsid interior [38]. Notably, the amino acid sequence alignment of HIV-1, SIV and FIV CA proteins predicts the conservation of this electropositive channel structure, which suggests that the nucleotide import mechanism driven by the hexameric CA structure may represent a general lentiviral strategy to regulate both viral copy DNA synthesis and core uncoating [38].

Despite their low sequence similarity, all the retroviral CA proteins are organized in two highly α-helical regions that fold independently of each other: an N-terminal domain (CA-NTD) and a C-terminal domain (CA-CTD) connected by a flexible linker [37,39–41]. Within the CA-CTD lies a 20-amino-acid motif, conserved across retroviruses, known as the major homology region (MHR) [42], which plays a crucial role in Gag assembly [43–47]. Several structural studies, mainly on the HIV-1 CA, have revealed that the assembly of retroviral particles follows a structural leitmotiv based on the formation of a hexagonal Gag lattice in which the CA-NTD organizes into hexameric rings connected by CA-CTD homodimers [17–20,40,48–50].

Despite being phylogenetically related, SIV and FIV exhibit distinctive features that reflect the evolutionary divergence between these lentiviruses, such as the receptor/coreceptor complex used for virus entry and the number and functions of their accessory proteins [3,51,52]. Given that the knowledge on the CA domains of the SIV and FIV Gag proteins is particularly scarce, we decided to investigate their functional equivalence. In a previous study, based on the characterization of chimeric SIVs carrying FIV CA-derived regions [52], we demonstrated that a chimeric SIV containing the FIV CA, p1, and NC sequences upstream of the first zinc-finger motif produces virions at levels similar to those of wild-type SIV. Moreover, we showed that the FIV CA-CTD is the minimal FIV CA region that can functionally substitute for its SIV counterpart in the context of SIV Gag [52].

In the present work, we extended our studies on the functional relationship between these lentiviral CA proteins by analyzing the ability to assemble into immature virus-like particles (VLPs) of FIV Gag proteins in which the CA domain is partially or totally replaced by its equivalent SIV region. In contrast to the phenotypes previously observed for the chimeric SIV Gag proteins, none of the FIV Gag chimeras is assembly-competent. Of note, all these chimeric Gag proteins interact with wild-type SIV Gag and are copackaged into VLPs, irrespective of the swapped CA-derived region.

## Materials and methods

### Plasmid constructs

The chimeric FIV *gag* genes were generated by replacing different sequences within the CA-p1-NC region of the molecular clone FIV-14 of the Petaluma isolate [53] with the equivalent sequences of the SIV_SMM-PBj_ *gag* gene [4]. The pcDNA-FIV*gag* plasmid coding for wild-type FIV Gag (nucleotides [nt] 628–1980 of the FIV-14 genome), which was used as the gene backbone to substitute the SIV CA-derived sequences for those of FIV, has been described previously [47]. The chimeric DNA constructs were generated by a PCR-based procedure similar to that recently reported [52] using the Q5 High-Fidelity DNA polymerase (New England BioLabs). To generate the FIV_SIVCA(NTD)_ chimera, the amplified DNA fragment corresponding to FIV MA-coding sequences (nt 628–1032) was ligated to the DNA coding for the SIV CA residues Pro1 to Asp151 (nt 1234–1686 of the SIV_SMM-PBj_ genome). The resulting fragment was then joined to the FIV *gag* region comprising Leu145-Leu222 of the FIV CA, p1, NC, and p2 (nt 1465–1980 of FIV-14). In the case of the chimeric FIV_SIVCA(CTD)_ *gag* gene, the FIV sequences coding for the MA and CA residues 1–142 (nt 628–1458) were first joined to the SIV region corresponding to CA residues Leu150-Met230 (SIV_SMM-PBj_ nt 1681–1923), and then ligated to the sequences encoding FIV p1 together with the downstream *gag* region (nt 1699–1980 of FIV-14). The FIV_SIVCA-SP1-NC(1–8)_ chimera was constructed by sequentially ligating the following PCR-amplified DNA fragments: the FIV MA-coding region of FIV-14 (nt 628–1032), a fragment coding for the entire SIV CA, the spacer peptide SP1 and the first 8 amino acids of NC (SIV_SMM-PBj_ nt 1234–1998), and an FIV-derived DNA fragment encoding residues Gly8 to Met66 of the NC domain and the downstream p2 sequences (FIV-14 nt 1747–1980). All the chimeric fragments were digested with BstXI and PflMI and substituted for the corresponding wild-type region in the pcDNA-FIV*gag* plasmid. The chimeric *gag* constructs were first screened by restriction mapping and then completely sequenced to verify the absence of fortuitous mutations and that the FIV-SIV sequences were joined in the correct reading frame. The construction of the pcDNA-SIV*gag* plasmid directing the expression of the wild-type SIV_SMM-PBj_ Gag precursor has been reported previously [46]. The chimeric SIV_FIVCA(CTD)_ *gag* gene, in which the SIV CA-CTD is replaced with that of FIV, was obtained by PCR amplification of the corresponding sequences using the proviral DNA genome SIV_FIVCA(CTD)_ as template [52] and cloned into the KpnI and EcoRI sites of the pcDNA3.1(+) vector (Invitrogen-Thermo Fisher Scientific).

For expression in *Escherichia coli*, the FIV *gag*_*SIVCA-SP1-NC(1–8)*_ chimeric gene was cloned into the EcoRV and SalI sites of the pET-30b (+) plasmid vector (Novagen). The construction of the pET-FIV*gag* plasmid, directing the synthesis of FIV Gag with an N-terminal histidine tag, has been described previously [54].

### Cell cultures and viruses

The COS-7 African green monkey kidney cell line (obtained from the American Type Culture Collection) was grown in Dulbecco’s modified Eagle’s medium (DMEM, GIBCO) supplemented with 10% fetal bovine serum (GIBCO) following standard protocols. The vaccinia virus vTF7-3 expressing the T7 RNA polymerase was kindly provided by Dr. B. Moss (NIAID, NIH, Bethesda, Maryland, USA).

### Viral protein expression in mammalian cell cultures and Western blotting

Expression of the wild-type FIV, SIV, and chimeric FIV-SIV *gag* genes was performed using the vaccinia T7 system essentially as previously described [46,47]. Briefly, confluent monolayers of COS-7 cells (grown in 35-mm-diameter dishes) were infected with the VT7-3 recombinant vaccinia virus at a multiplicity of 10 for 1 h at 37°C, then washed twice with DMEM, and transfected with the plasmid DNAs using Lipofectamine 2000 (Invitrogen-Thermo Fisher Scientific). In the case of the coexpression of each of the chimeric FIV Gag proteins with either wild-type FIV or SIV Gag, initial cotransfection experiments were carried out to determine the appropriate mass ratio of the corresponding expression plasmids that ensures comparable intracellular levels of both Gag proteins. Thirty hours post-transfection, cells were washed twice with ice-cold phosphate-buffered saline (PBS) and lysed at 4°C in lysis buffer (50 mM Tris-HCl [pH 8.0], 150 mM NaCl, 1% Nonidet P-40, 0.1% sodium dodecyl sulfate [SDS], 0.5% sodium deoxycholate, and Protease Inhibitor Cocktail [Roche]). The culture supernatants from the infected/transfected cells were filtered through 0.45-μm-pore-size syringe filters and VLPs were pelleted from the clarified supernatants by ultracentrifugation (100,000 x*g*, 90 min, 4°C) through a 20% (w/v in PBS) sucrose cushion as we have previously reported [46,52]. Viral proteins in the cell and VLP lysates were resolved on SDS-9% or -10% polyacrylamide gels, blotted onto nitrocellulose membranes, and analyzed by Western blotting coupled with an enhanced chemiluminescence assay (Western Lightning ECL Pro, PerkinElmer). The following antibodies were used to detect the wild-type and chimeric Gag proteins: the anti-FIV CA monoclonal antibody (MAb) PAK3-2C1 (NIH AIDS Reagent Program, Division of AIDS, NIAID, NIH); an anti-FIV MA mouse polyclonal antibody prepared in our laboratory [47]; the anti-SIV CA MAb KK60 (MRC AIDS Directed Program); the MAb AG3.0 to HIV-1 p24 (NIH AIDS Reagent Program) that recognizes the CA-NTD epitope SPRTLNA (residues15-21 in SIV CA) conserved among HIV-1, HIV-2, and SIV_SMM/mac_ [55]; and a mouse anti-SIV MA polyclonal serum obtained in our laboratory [13]. Horseradish peroxidase (HRP)-conjugated anti-mouse immunoglobulin (Cayman Chemical) was used as secondary antibody.

### Expression in *E*. *coli* and purification of recombinant proteins

Histidine-tagged Gag proteins were produced in *E*. *coli* BL21 (DE3) and purified by affinity chromatography following procedures reported previously [46,54]. Protein concentrations were estimated as we have already described [13,46]. Briefly, recombinant FIV Gag proteins were run in parallel on SDS-polyacrylamide gels with known amounts of standard bovine serum albumin. Gels were stained with Coomassie G-250 SimplyBlue SafeStain (Invitrogen) and the intensity of the protein bands was compared by densitometry. For the *in vitro* assembly reactions, an aliquot of protein extracts was further treated with DNase I (5 units; Promega) and RNase A (50 μg/ml; Sigma-Aldrich) to remove nucleic acids before purification by affinity chromatography. Potential contamination of purified Gag proteins with nucleic acids was evaluated by spectrophotometry measuring the A_260_/A_280_ ratio as well as by agarose gel electrophoresis after phenol extraction and ethanol precipitation of protein samples.

### *In vitro* assembly of FIV Gag proteins

The protocols for the synthesis and purification of the RNA corresponding to the FIV packaging signal (nt 216–947 of FIV-14; referred to here as FIV R-U5-MA) have been published previously [26,54].

Purified recombinant histidine-tagged FIV Gag_SCA-SP1-NC(1–8)_ and wild-type FIV Gag proteins stored at -80°C were thawed on ice, first centrifuged at 16,000 x*g* for 20 min, and the resulting supernatants were then used in the *in vitro* assembly reactions essentially as we have described previously [46,54]. The recombinant Gag proteins (5 μg) were incubated for 3 h at 37°C in 25-μl reactions containing 50 mM Tris-HCl (pH 8.0), 150 mM NaCl, 5 mM dithiothreitol, 10 mM ZnCl_2_, 500 ng of *in-vitro*-transcribed FIV R-U5-MA RNA, and 100 units of recombinant RNasin ribonuclease inhibitor (Promega), and the assembly reactions were then centrifuged for 1 h in an Eppendorf microcentrifuge at 16,000 x*g* at 4°C to separate the particulate assembled structures from the unassembled molecules [46,54]. The Gag proteins in the supernatant and pellet fractions were resolved by SDS-polyacrylamide gel electrophoresis (SDS-PAGE), blotted onto nitrocellulose membranes, and detected by immunoblotting with the serum specific for the FIV MA protein.

## Results

### Rationale for the construction of FIV *gag* genes carrying SIV CA-coding sequences

**Fig 1:**
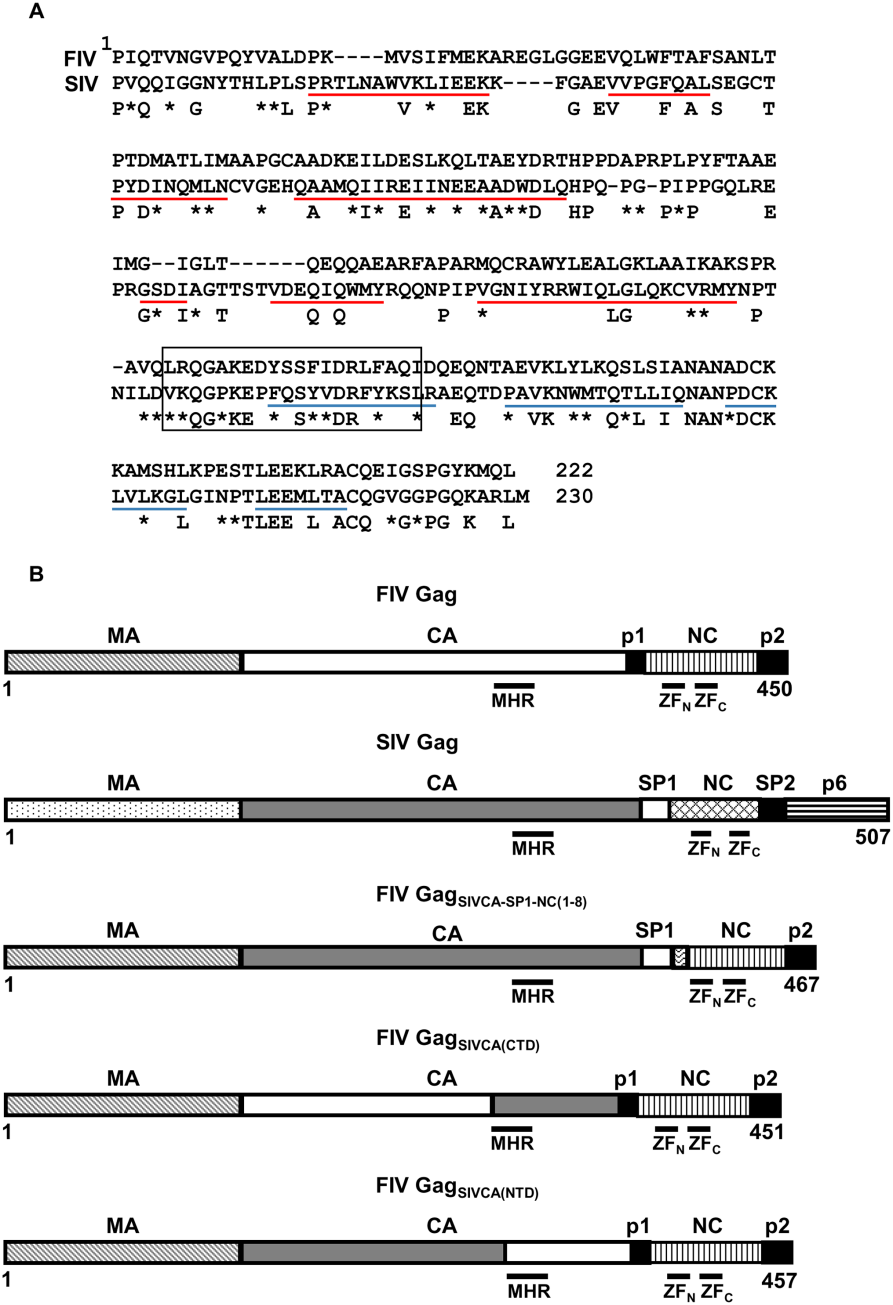
Chimeric FIV Gag polyproteins generated to investigate the functional relationship between the FIV and SIV CA domains. (A) Alignment of the amino acid sequences of the FIV (Petaluma isolate) and SIV (molecular clone SIV_SMM_PBj1.9) CA domains of Gag. The residue numbering is relative to the first amino acid (Pro) of the mature CA proteins. Identical amino acids present at the same position in both CA proteins are highlighted. Asterisks denote conservative amino acid substitutions. The open box corresponds to the MHR of the CA proteins. The NTD and CTD α-helices found in the HIV-1 CA structure [40,56] are indicated with red and blue lines, respectively, under the alignment. (B) Schematic diagram of the FIV Gag chimeras analyzed in this work. The organization of the wild-type FIV and SIV Gag precursors is depicted at the top showing the structurally conserved domains (MA, CA, and NC), the C-terminal domains (p2 in FIV Gag; p6 in SIV Gag), as well as the spacer peptides (p1 in FIV Gag; SP1 and SP2 in SIV Gag). The positions of the MHR in CA, and the N-terminal (ZF_N_) and C-terminal (ZF_C_) zinc-finger motifs in the NC domains of both FIV and SIV Gag proteins are indicated: The numbers refer to the length of each of the chimeric FIV Gag polyproteins, with residue 1 being the initiator methionine in Gag.

Recently, we showed that chimeric SIVs in which either the FIV CA-CTD alone, or the FIV CA-p1 region along with the first nine residues of FIV NC are substituted for the equivalent region of SIV_SMM-PBj_ assemble into virions that incorporate the Env glycoprotein, package wild-type levels of viral genomic RNA and contain a functional reverse transcriptase; yet these chimeric SIVs are non-infectious due to a defect at a post-entry step [52]. Based on these findings, and given the biological relevance of the CA domain in Gag assembly, we aimed to investigate further the functional relationship between the lentiviral SIV and FIV CA proteins, which share 51% overall amino acid sequence similarity (Fig 1A), by examining how the assembly of immature FIV Gag particles is modulated by the SIV CA. To this end, we generated FIV Gag polyproteins carrying different SIV CA-derived sequences (Fig 1B). The chimeras FIV *gag*_*SIVCA(NTD*)_ and FIV *gag*_*SIVCA(CTD)*_ were constructed to study the functional compatibility of the SIV CA-NTD and CA-CTD, respectively, in the context of the FIV Gag precursor. In addition, the FIV *gag*_*SIVCA-SP1-NC(1–8)*_ gene was generated to evaluate whether the SIV module comprising the entire CA domain and the C-terminally adjacent sequences extending from SP1 up to the amino acid residue 8 of NC are able to drive VLP assembly in the context of the remaining FIV Gag domains.

### Assembly phenotype of chimeric FIV Gag_SIVCA-SP1-NC(1–8)_ in mammalian cells

**Fig 2:**
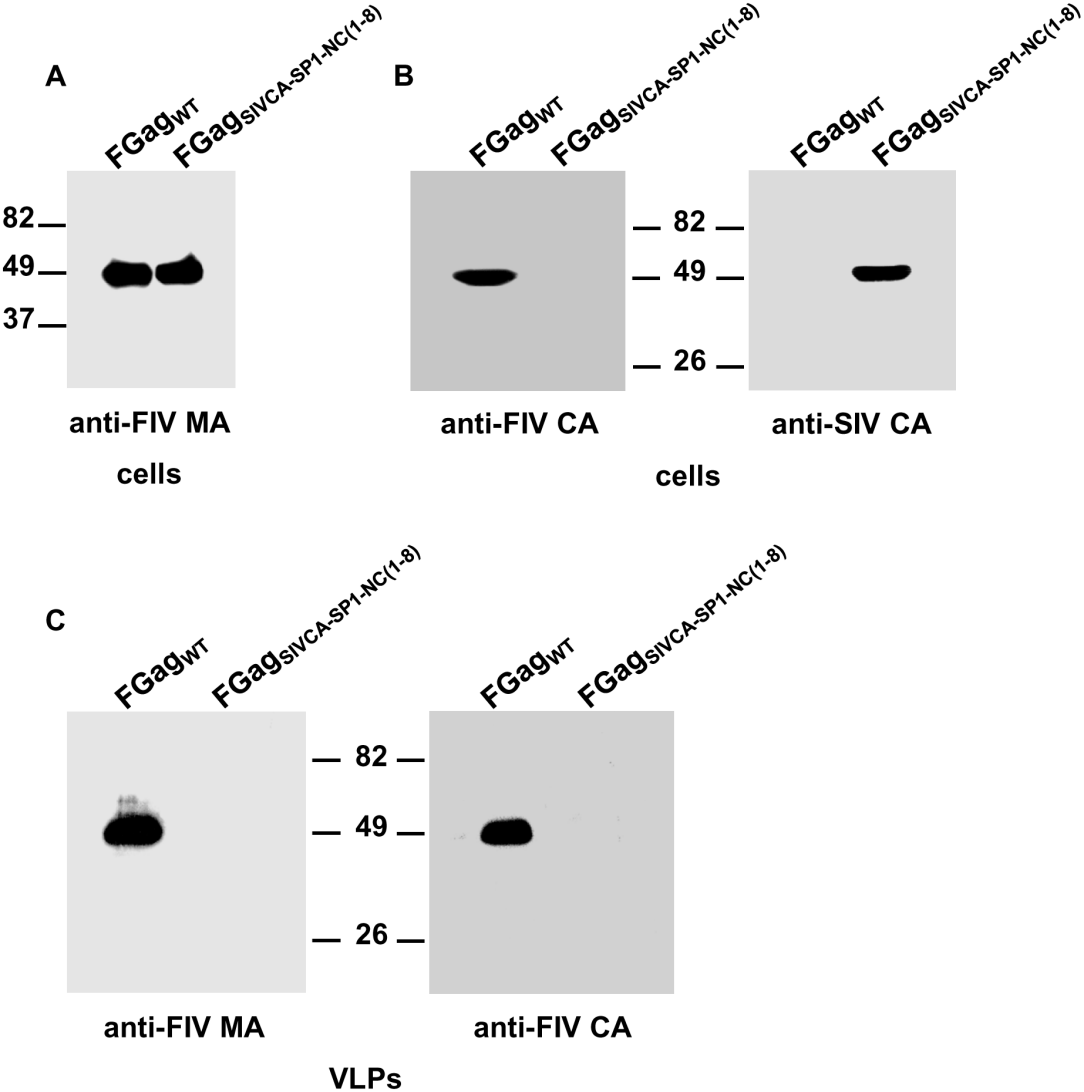
Effect of substituting the SIV CA-SP1-NC_(1–8)_ region for its FIV equivalent on the production of extracellular VLPs. COS-7 cells infected with the vTF7-3 recombinant vaccinia virus were transfected with the plasmid directing the expression of wild-type FIV Gag (FGag_WT_) or FIV Gag_SIVCA-SP1-NC(1–8)_ (FGag_SIVCA-SP1-NC(1–8)_). Thirty hours post-transfection, cells were harvested and VLPs were purified from the culture supernatants as explained in Materials and Methods. Cell (A and B) and VLP (C) lysates were analyzed for the presence of Gag proteins by Western blotting using the anti-FIV MA serum (A and C), the MAb specific for the FIV CA-CTD (B and C; indicated as anti-FIV CA), or the anti-SIV CA MAb KK60 directed to the SIV CA MHR to unambiguously detect the FIV Gag_SIVCA-SP1-NC(1–8)_ chimera (B; indicated as anti-SIV CA). Numbers indicate the positions of the molecular weight standards (in kDa). Results shown are representative of three independent experiments.

To investigate whether substituting the SIV *gag* region comprising the CA, SP1, and the first eight amino acids of the NC protein for the equivalent FIV sequences confers assembly competence to the chimeric FIV Gag precursor, the plasmids encoding the FIV Gag_SIVCA-SP1-NC(1–8)_ chimera, or, as control, wild-type FIV Gag were transfected in parallel into COS-7 cells previously infected with the recombinant vaccinia virus expressing the T7 RNA polymerase. Cell and VLP lysates were resolved by SDS-PAGE, and subjected to Western blotting using a polyclonal anti-FIV MA serum to detect both the wild-type and chimeric FIV Gag proteins, or, MAbs specific for either the SIV CA, or the FIV CA proteins to distinguish FIV Gag_SIVCA-SP1-NC(1–8)_ from wild-type FIV Gag. As shown in Fig 2, the chimeric Gag_SIVCA-SP1-NC(1–8)_ polyprotein was found to be assembly-incompetent although it was expressed at wild-type levels. This result was somewhat unexpected, since this FIV Gag chimera contains the complete heterologous SIV CA-SP1 assembly unit known to be crucial for immature particle formation in HIV-1 and SIV [14–16,20,46].

### Assembly phenotype of chimeric FIV Gag_SIVCA-SP1-NC(1–8)_ *in vitro*

We have previously demonstrated that the full-length FIV Gag protein expressed in bacteria and purified by affinity chromatography is capable of assembling *in vitro* into spherical particles that are morphologically similar to the VLPs produced upon expression of FIV Gag in the vaccinia system [54].

**Fig 3:**
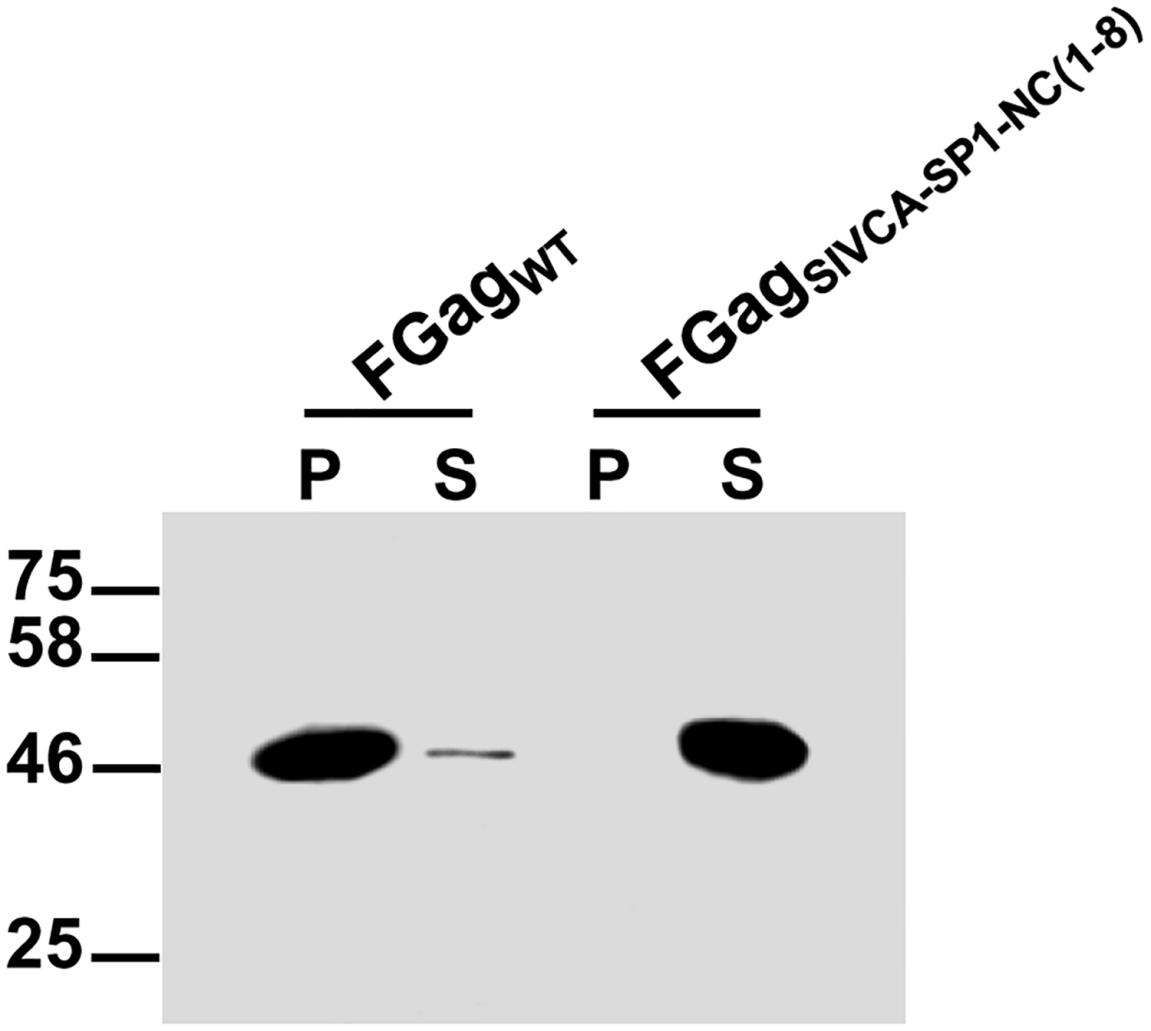
*In vitro* assembly reaction for recombinant FIV Gag_SIVCA-SP1-NC(1–8)_. The purified His-FIV Gag_SIVCA-SP1-NC(1–8)_ (FGag_SIVCA-SP1-NC(1–8)_) and His-FIV Gag_wild-type_ (FGag_WT_) proteins were incubated in parallel under the conditions described in Materials and Methods and the assembly mixtures were separated by centrifugation into the pellet (P) and supernatant (S) fractions which were then subjected to SDS-PAGE followed by Western blotting using the anti-FIV MA serum. Results shown are representative of three independent experiments.

The data described in the former section, together with the results of our precedent work showing that a chimeric SIV carrying the FIV CA-p1-NC_(1–9)_ region produces virions as efficiently as wild-type SIV [52], prompted us to address whether the recombinant FIV Gag_SIVCA-SP1-NC(1–8)_ precursor expressed in *E*. *coli* is able to self-assemble *in vitro* under chemically defined conditions. The purified histidine-tagged wild-type FIV Gag and FIV Gag_SIVCA-SP1-NC(1–8)_ proteins were incubated in parallel under the conditions described in Materials and Methods, followed by centrifugation of the assembly reactions to separate the pelletable Gag-made particles (P fraction) from the unassembled Gag molecules that remain in the supernatant (S fraction). The P and S fractions from the assembly reactions of both wild-type and chimeric FIV Gag were then analyzed by Western blotting using the anti-FIV MA serum. As expected, most of the wild-type FIV Gag protein was found in the P fraction (Fig 3). By contrast, FIV Gag_SIVCA-SP1-NC(1–8)_ partitioned with the S fraction (Fig 3), confirming that this chimera is incapable of self-assembling into multimeric complexes.

### Ability of chimeric FIV Gag_SIVCA-SP1-NC(1–8)_ to interact with wild-type FIV or SIV Gag

**Fig 4:**
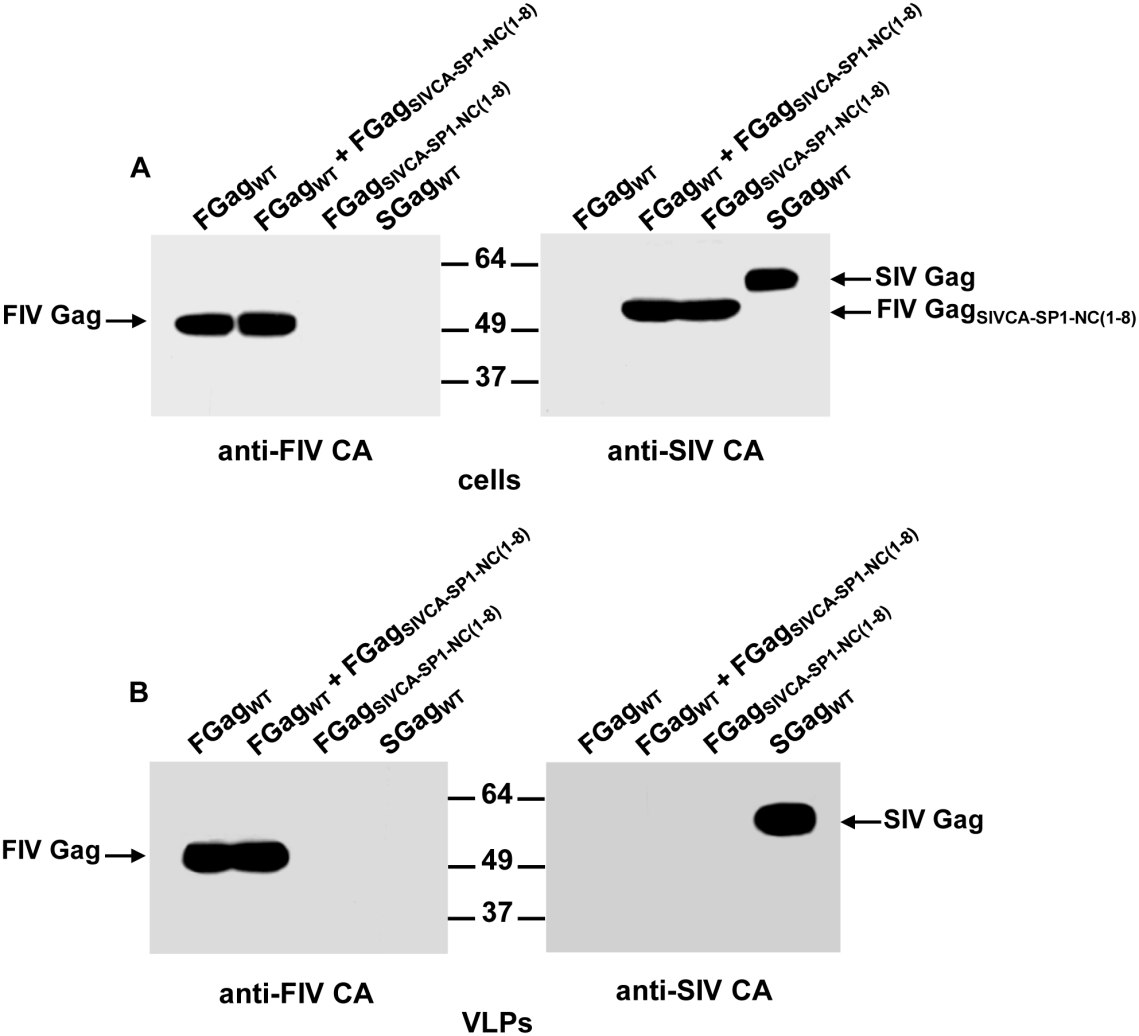
Analysis of the association capacity of FIV Gag_SIVCA-SP1-NC(1–8)_ with wild-type FIV Gag. COS-7 cells infected with the vTF7-3 recombinant vaccinia virus were transfected with the plasmid expressing FIV Gag_SIVCA-SP1-NC(1–8)_ (FGag_SIVCA-SP1-NC(1–8)_) or cotransfected with the constructs coding for wild-type FIV Gag (FGag_WT_) and FIV Gag_SIVCA-SP1-NC(1–8)_. As controls, cells were transfected in parallel with the plasmids encoding either wild-type FIV Gag or wild-type SIV Gag (SGag_WT_). Protein blots of cell (A) and VLP (B) lysates were probed with the antibodies specific for the FIV or SIV CA-CTD (indicated in the panels as anti-FIV CA and anti-SIV CA, respectively). The relative mobilities of both wild-type FIV Gag and SIV Gag as well as that of the chimeric FIV Gag protein are shown. Numbers refer to the positions of the molecular weight standards (in kDa). Results shown are representative of three independent experiments.

**Fig 5:**
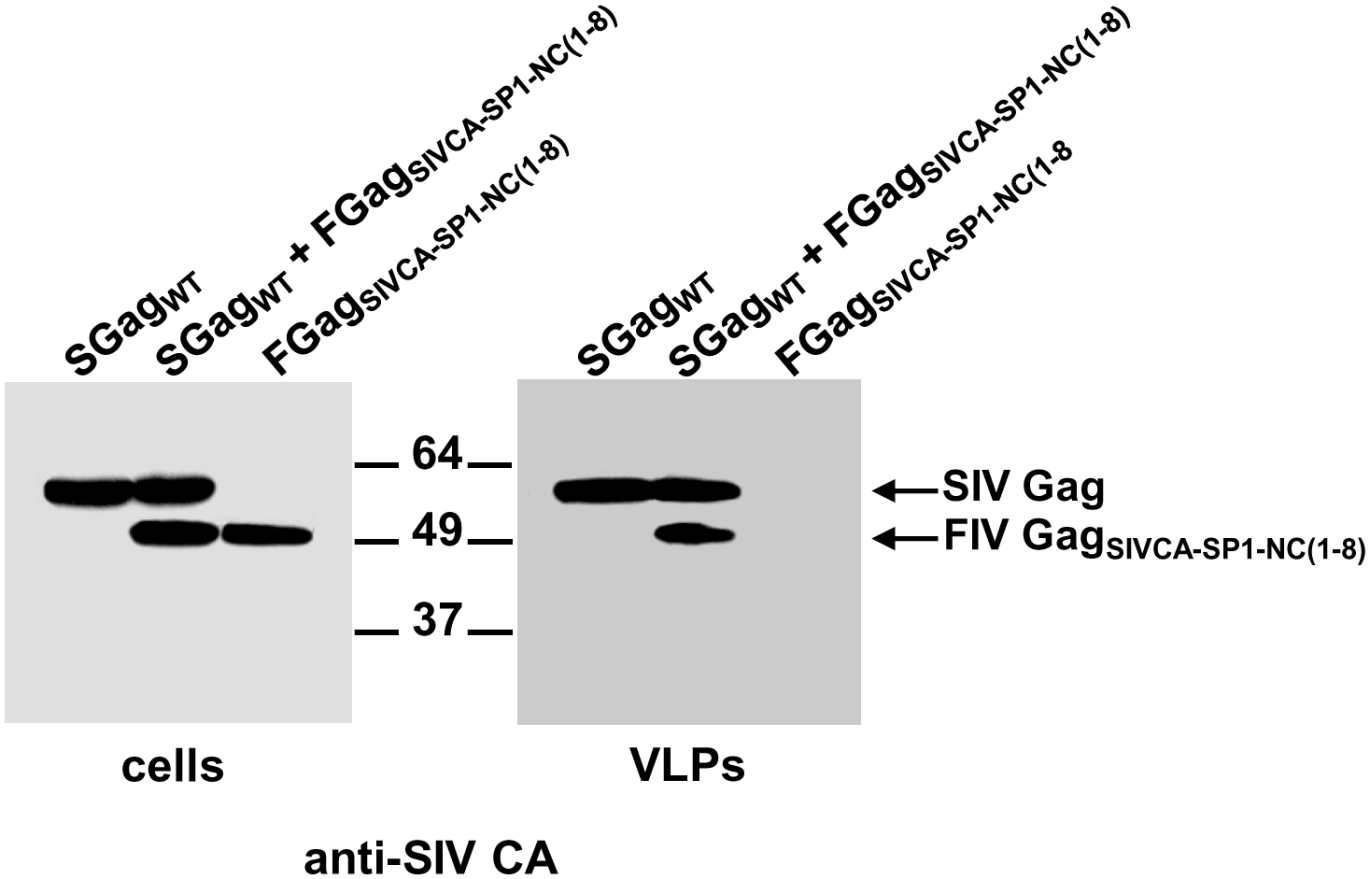
Ability of the chimeric FIV Gag_SIVCA-SP1-NC(1–8)_ to interact with wild-type SIV Gag in cell cultures. COS-7 cells infected with the vTF7-3 recombinant vaccinia virus were transfected with plasmids expressing either wild-type SIV Gag (SGag_WT_) or FIV Gag_SIVCA-SP1-NC(1–8)_ (FGag_SIVCA-SP1-NC(1–8)_), or cotransfected with both plasmids. The Gag proteins in the cell (A) and VLP (B) lysates were detected by Western blotting using the anti-SIV CA MAb KK60. The relative mobilities of the wild-type and chimeric Gag proteins are shown, as are the positions of the molecular weight markers (in kDa). Results shown are representative of three independent experiments.

We next examined whether wild-type FIV or SIV Gag proteins can rescue the chimeric FIV Gag_SIVCA-SP1-NC(1–8)_ into extracellular particles as the result of protein interactions between the chimeric Gag protein and the wild-type counterparts from either FIV or SIV. Indeed, we have extensively made use of this strategy to map the self-interaction domains in both SIV and FIV Gag precursors [46,47]. To this end, cells were transfected with the plasmid coding for FIV Gag_SIVCA-SP1-NC(1–8)_ together with that encoding wild-type FIV Gag (Fig 4) or wild-type SIV Gag (Fig 5). Analysis of both cell and VLP lysates from these coexpression experiments showed that FIV Gag_SIVCA-SP1-NC(1–8)_ was only present in the VLPs formed by wild-type SIV Gag (compare Figs 4 and 5), which indicates that this chimeric FIV Gag precursor is only capable of establishing Gag-Gag interactions with wild-type SIV Gag.

### Analysis of the assembly capacity of FIV Gag_SIVCA(CTD)_

**Fig 6:**
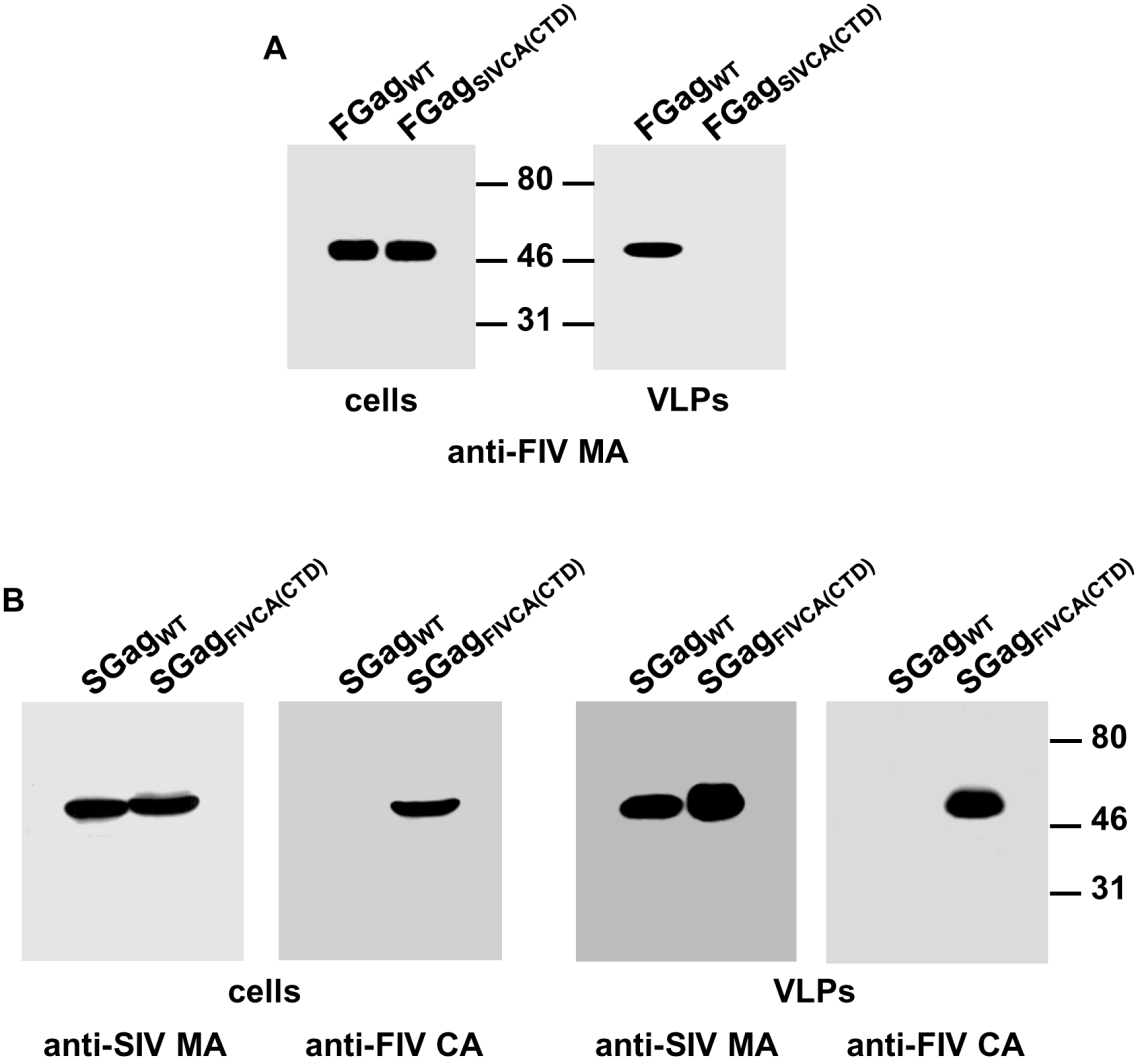
Comparative analysis of the effect of exchanging the FIV and SIV CA-CTD on chimeric Gag assembly. COS-7 cells infected with the vTF7-3 recombinant vaccinia virus were transfected with plasmids directing the synthesis of wild-type FIV Gag (FGag_WT_) or FIV Gag_SIVCA(CTD)_ (FGag_SIVCA(CTD)_) (A), and, in parallel, with plasmids coding for wild-type SIV Gag (SGag_WT_) or SIV Gag_FIVCA(CTD)_ (SGag_FIVCA(CTD)_) (B). Cell and VLP lysates were analyzed for the presence of Gag proteins by Western blotting using the anti-FIV MA serum (A), or the anti-SIV MA serum and the anti-FIV CA MAb (B). Numbers indicate the positions of the molecular weight standards (in kDa). Results shown are representative of three independent experiments.

Given that retroviral CA proteins are organized in independent folding units, we first examined whether the SIV CA-CTD can functionally replace its FIV CA counterpart. As shown in Fig 6A, the chimeric FIV Gag_SIV CA(CTD)_ exhibited steady-state levels similar to those of wild-type FIV Gag; however, it was incapable of assembling into VLPs. This result is in marked contrast to our previous finding that SIV_FIVCA(CTD)_, a chimeric SIV carrying only the FIV CA-CTD, efficiently assembles into virions [52]. To rule out the possibility that the assembly-competent phenotype exhibited by SIV_FIVCA(CTD)_ might be due to its expression in the context of the viral genome, and therefore be influenced by the viral protease-mediated processing of the chimeric Gag precursor [52], we examined the ability of the SIV Gag_FIVCA(CTD)_ polyprotein to assemble into immature particles by means of the same vaccinia virus/T7 expression strategy as that used in this paper for the chimeric FIV Gag polyproteins. In agreement with our results obtained with the proviral genomes [52], the chimeric SIV Gag_FIVCA(CTD)_ precursor assembles into immature VLPs as efficiently as wild-type SIV Gag (Fig 6B).

### Ability of chimeric FIV Gag_SIVCA(CTD)_ to interact with wild-type FIV or SIV Gag

**Fig 7:**
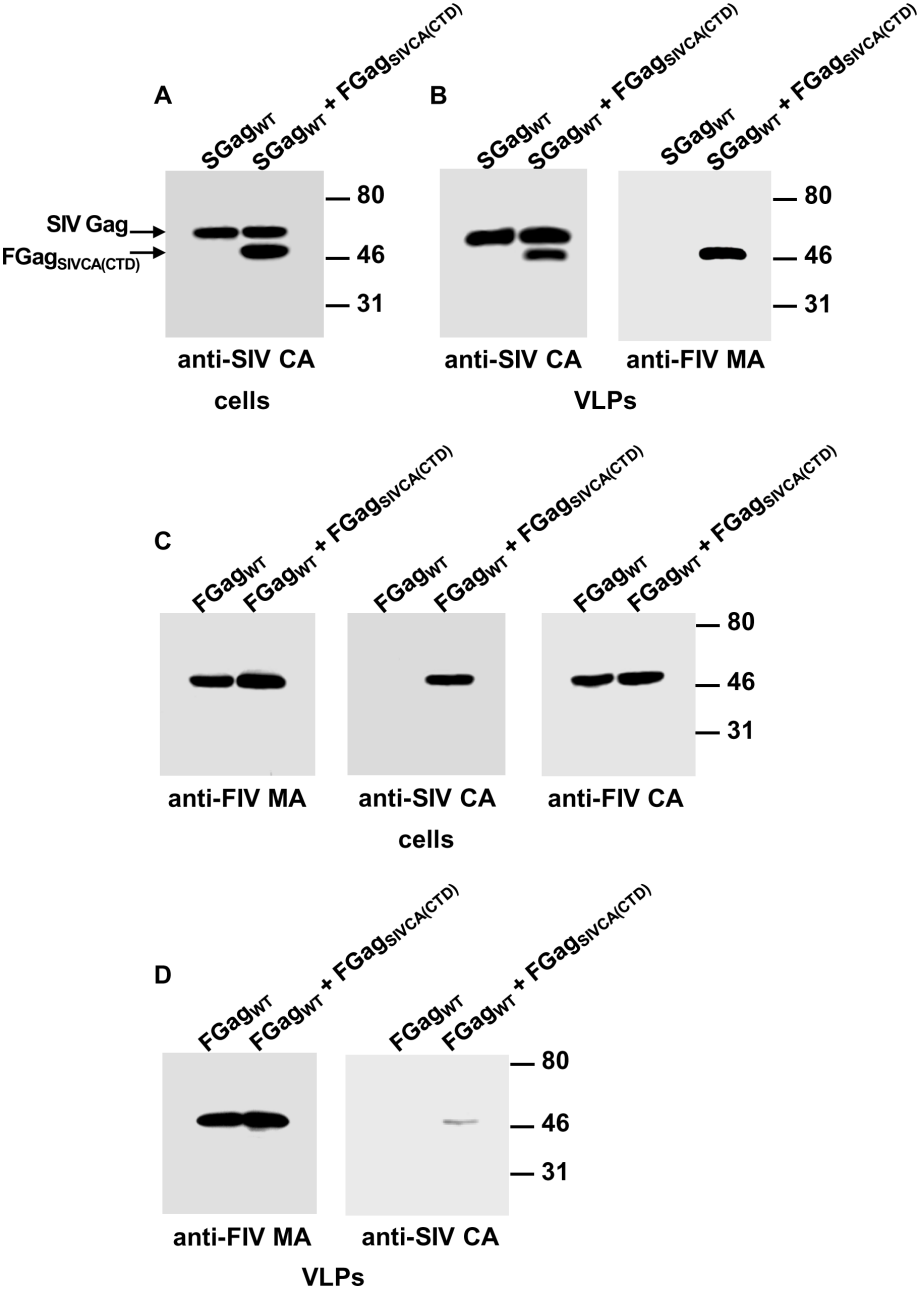
Interaction of the chimeric FIV Gag_SIVCA(CTD)_ polyprotein with wild-type SIV and FIV Gag. COS-7 cells infected with the vTF7-3 vaccinia virus were transfected with the plasmid coding for wild-type SIV Gag (SGag_WT_) alone, or together with that expressing FIV Gag_SIVCA(CTD)_ (FGag_SIVCA(CTD)_) (A and B). In parallel, cells were transfected with the construct coding for wild-type FIV Gag (FGag_WT_) alone or together with the plasmid expressing FIV Gag_SIVCA(CTD)_ (C and D). Cell- and VLP-associated proteins were resolved by SDS-PAGE and the Gag proteins were visualized by Western blotting using the anti-FIV MA serum, or the MAbs targeted to the FIV or SIV CA-CTD (indicated in the panels as anti-FIV CA and anti-SIV CA, respectively). Numbers refer to the positions of the molecular weight standards (in kDa). Results shown are representative of three independent experiments.

We compared the capacity of FIV Gag_SIVCA(CTD)_ to associate with either wild-type FIV or SIV Gag and be copackaged into VLPs. Analysis by Western blotting of both cell and VLP lysates of COS-7 cells expressing the FIV Gag_SIVCA(CTD)_ precursor together with wild-type SIV Gag (Fig 7A and 7B) or wild-type FIV Gag (Fig 7C and 7D) demonstrated that the chimeric FIV Gag_SIVCA(CTD)_ is rescued into extracellular VLPs by both wild-type SIV and FIV Gag proteins, albeit to a significantly lesser extent in the latter case.

### Analysis of the FIV Gag_SIVCA(NTD)_ chimera

**Fig 8:**
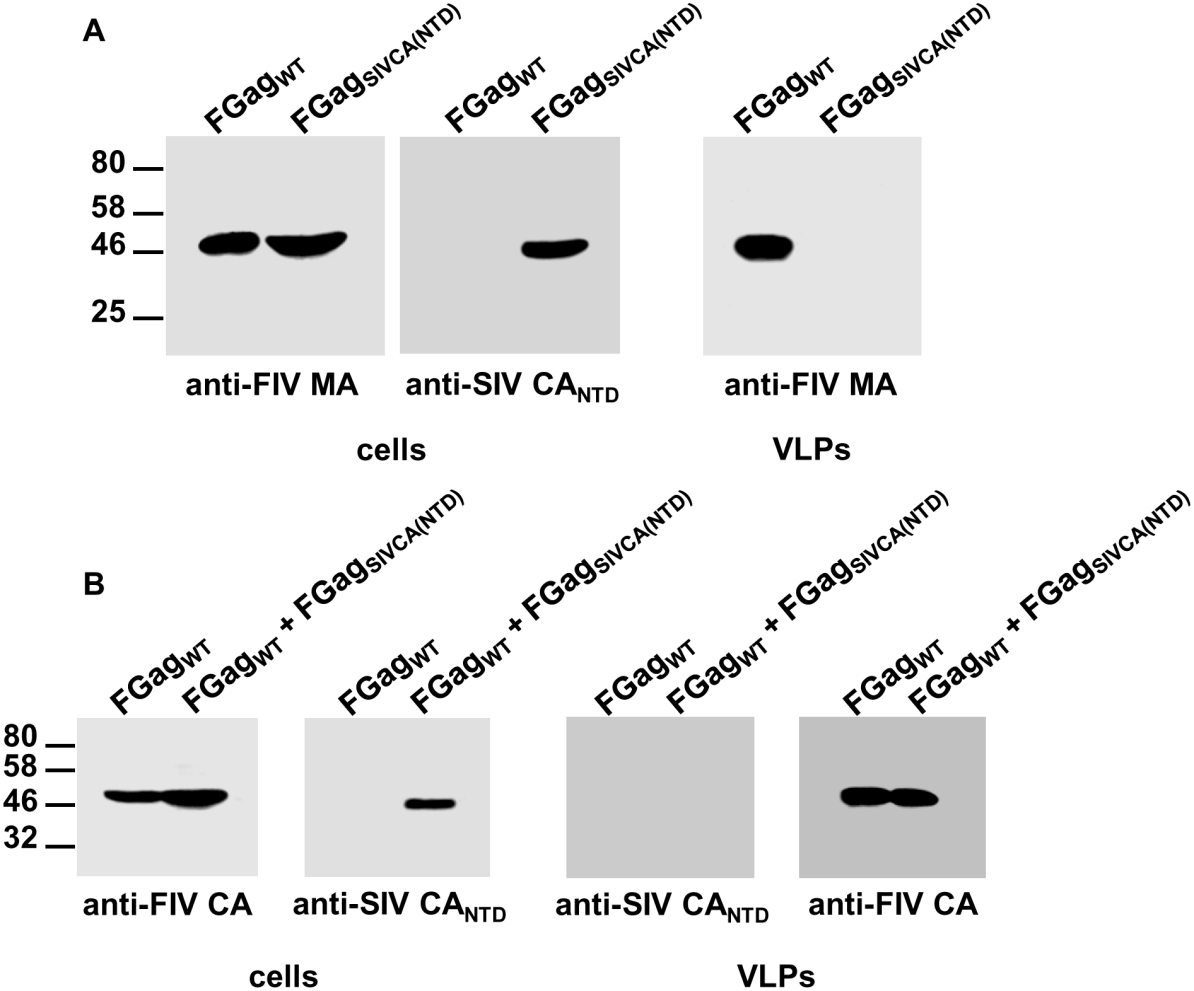
Phenotypic characterization of the FIV Gag_SIVCA(NTD)_ chimera. COS-7 cells infected with the vTF7-3 vaccinia virus were transfected with the plasmids expressing wild-type FIV Gag (FGag_WT_), or FIV Gag_SIVCA(NTD)_ (FGag_SIVCA(NTD)_) (A), or cotransfected with both plasmids (B). Cell- and VLP-associated proteins were detected by Western blotting using the anti-FIV MA serum, the MAb directed against the FIV CA-CTD (indicated as anti-FIV CA), or the MAb specific for the SIV CA-NTD. Numbers indicate the positions of the molecular weight standards (in kDa). Results shown are representative of three independent experiments.

**Fig 9:**
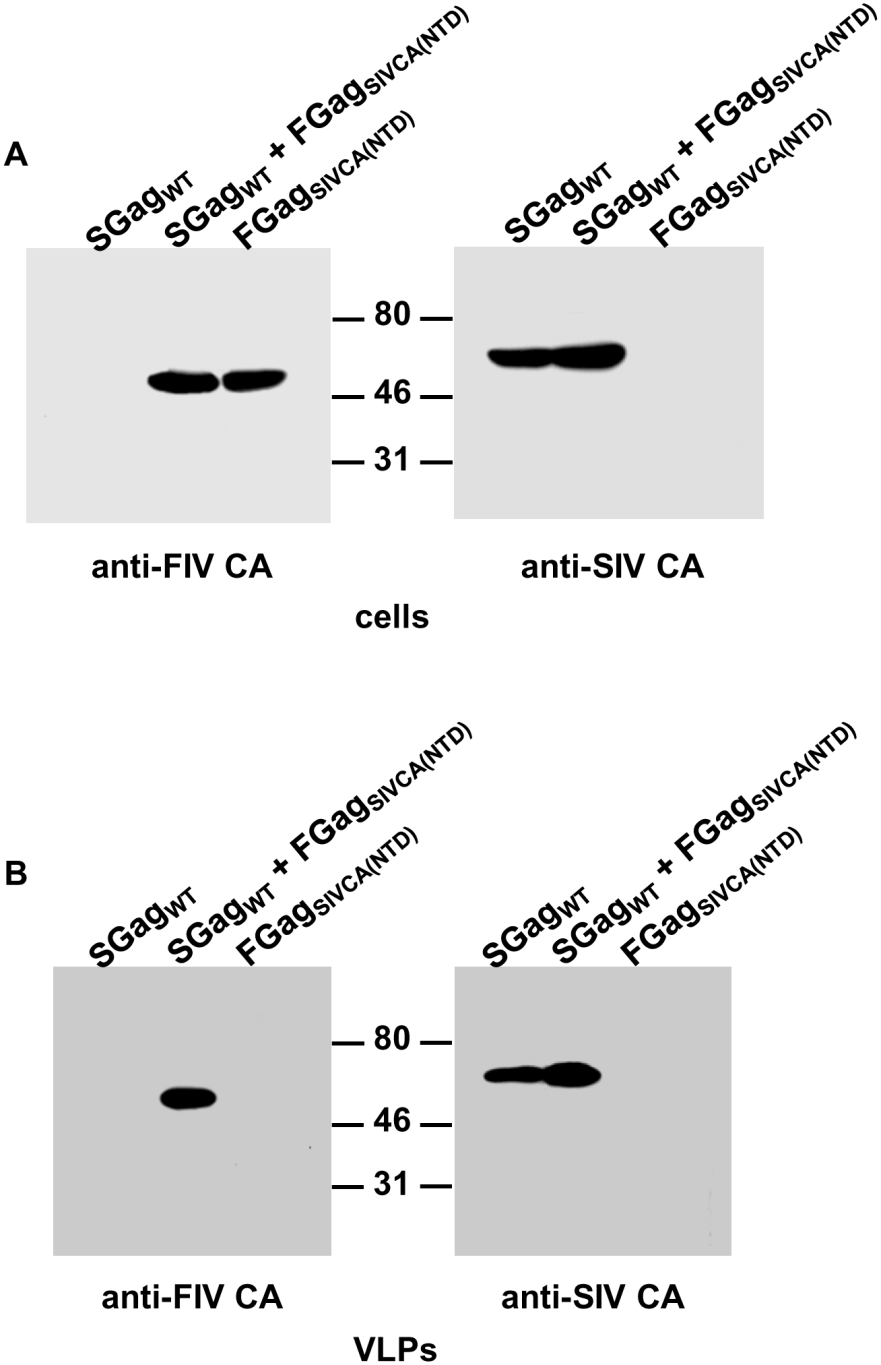
Interaction of FIV Gag_SIVCA(NTD)_ with wild-type SIV Gag. Cells infected with the vTF7-3 vaccinia virus were transfected with the plasmids expressing wild-type SIV Gag (SGag_WT_), FIV Gag_SIVCA(NTD)_ (FGag_SIVCA(NTD)_), or cotransfected with both plasmids. Cell and VLP lysates were analyzed for the presence of the wild-type SIV or chimeric Gag proteins by Western blotting using the MAbs directed to the SIV or FIV CA-CTD. Numbers indicate the positions of the molecular weight standards (in kDa). Results shown are representative of three independent experiments.

We also investigated whether the FIV CA-NTD can be functionally replaced by the equivalent domain of SIV CA. Analysis of the purified particulate fraction from the supernatant of cells expressing FIV Gag_SIVCA(NTD)_ revealed that it is incapable of assembling into VLPs (Fig 8A). Furthermore, wild-type FIV Gag cannot rescue FIV Gag_SIVCA(NTD)_ into VLPs (Fig 8B). In these Western blotting experiments, we used a MAb directed to the SIV CA-NTD to unequivocally distinguish the chimeric polypeptide from wild-type FIV Gag. By contrast, FIV Gag_SIVCA(NTD)_ is capable of establishing protein-protein interactions with wild-type SIV Gag, as evidenced by its presence in the VLP fraction from cells coexpressing this chimeric Gag protein with the wild-type SIV Gag precursor (Fig 9).

## Discussion

The assembly of immature lentivirus particles is a complex process that involves the coordination of Gag multimerization at the plasma membrane of the infected cell with the selective interplay between Gag molecules and viral and cellular components, which finally results in the formation of spherical biological structures that bud into the extracellular medium. During the assembly process, the CA domain plays a major role by establishing the protein-protein interactions that drive Gag multimerization into particles. Based on this concept, and to gain further insight into the functional equivalence between the lentiviral SIV and FIV CA domains, we investigated whether substitution of SIV CA-derived regions for their FIV CA counterparts can confer assembly-competence to the resulting FIV Gag chimeras. The phenotypic analyses of the chimeric FIV Gag precursors showed that all are assembly-incompetent, which indicates that none of these proteins adopts the correct conformation necessary to make the homomeric Gag-Gag contacts involved in immature VLP formation. Interestingly, despite being assembly-defective, all the FIV Gag chimeras interact with wild-type SIV Gag and are copackaged into SIV Gag VLPs, irrespective of the swapped CA region. By contrast, only the chimera carrying the SIV CA-CTD is rescued by wild-type FIV Gag into VLPs. Nonetheless, the extent to which wild-type FIV Gag associates with FIV Gag_SIVCA(CTD)_ is not comparable to the high efficiency with which wild-type SIV Gag interacts with all the FIV Gag chimeras.

These results are in marked contrast to our previous studies with chimeric SIVs demonstrating that the FIV CA-CTD alone or the FIV region encompassing the CA, p1 and the first nine residues of NC can functionally substitute for their SIV counterparts. Moreover, these SIV chimeras produce virions exhibiting a mature and stable FIV CA protein, and contain wild-type levels of viral genome RNA and reverse transcriptase [52]. The assembly competence of the chimeric SIVs versus the inability of the FIV Gag chimeras to assemble into VLPs cannot be attributed to the presence of an active protease in the former set of Gag chimeras, which leads to viral particle maturation, since we show here that the SIV Gag_FIVCA(CTD)_ precursor exhibits the same assembly-competent phenotype as that of the chimeric virus in which processing of SIV Gag_FIVCA(CTD)_ is regulated by the virus-encoded protease.

Comparison of the results presented here with those obtained from the characterization of chimeric SIVs [52] allows us to draw the following conclusions:

First, the pair of heterologous domains FIV CA-NTD/SIV CA-CTD is nonfunctional in either the SIV or FIV Gag context. Indeed, we have shown previously that the chimeric virus SIV_FIVCA(NTD)_ is assembly-incompetent [52]. Nevertheless, the sole presence of the SIV CA-CTD in the FIV Gag_SIVCA(CTD)_ polyprotein is sufficient to mediate the efficient interaction between this chimera and wild-type SIV Gag. Furthermore, FIV Gag_SIVCA(CTD)_ is the only chimera capable of associating with wild-type FIV Gag and being rescued into VLPs, albeit with an efficiency significantly lower than that with SIV Gag.

Second, the combination of the SIV CA-NTD/FIV CA-CTD modules is only functional in the context of the SIV Gag polyprotein, even though the FIV CA-CTD region in the chimeric SIV_FIVCA(CTD)_ is N- and C-terminally flanked by heterologous SIV sequences [52].

Third, it is worth commenting on the implications of the defective phenotype of the FIV Gag chimera that bears the SIV CA-NTD but conserves the FIV CA-CTD. Based on our demonstration that the FIV CA-CTD has an intrinsic ability to dimerize in solution [52], which is supported by recent structural data showing that the crystallographically characterized CTD dimerization interface of HIV-1 CA [45,57] is also present in FIV CA [58], it can be concluded that although the FIV CA-CTD dimerization interface is preserved in FIV Gag_SIVCA(NTD)_, it is not sufficient to confer to the chimeric Gag either the ability to assemble into VLPs or to be rescued in *trans* by wild-type FIV Gag. Furthermore, our data suggest that the heterologous SIV CA-NTD in the context of the FIV Gag backbone has a detrimental effect on FIV Gag assembly and may affect the quaternary structure that the chimeric Gag needs to adopt to multimerize into particles.

Fourth, only SIV Gag allows the replacement of its entire CA together with the immediately adjacent spacer peptide and the first NC residues by the equivalent region of FIV [52]. In this regard, the inability of FIV Gag_SIVCA-SP1-NC(1–8)_ to assemble into VLPs either *in vivo* or *in vitro* argues against the notion that Gag precursors bearing a CA-NTD and a CA-CTD from the same CA protein are expected to invariably be assembly-competent, as suggested by Ako-Adjei *et al*. [59].

Fifth, the fact that swapping of CA regions between SIV and FIV Gag polyproteins is reciprocally non-equivalent underscores the relevance of the other Gag domains apart from CA for the organization and/or preservation of the Gag lattice during particle assembly.

Importantly, we have previously demonstrated that replacement of the FIV MA domain by that of SIV results in a chimeric FIV that not only assembles into virions but replicates in feline lymphoid cells with wild-type kinetics [8]. The fact that the SIV MA is biologically active in the context of FIV Gag whereas all the SIV CA-derived sequences tested here have a deleterious effect on FIV Gag assembly is likely related to two non-mutually exclusive phenomena: (a) the different functions that the MA and CA domains perform during membrane targeting, membrane binding and multimerization of the Gag molecules; (b) the temporal order in which the functionally distinct surfaces of MA, CA-NTD and CA-CTD act during the formation of the immature particles. This notion is supported by the model proposed by Robinson *et al*. [60] for the temporal and spatial order in which each of the HIV-1 Gag domains participates in the interactions that mediate particle assembly.

In conclusion, the contrasting assembly behavior between the previously characterized chimeric SIVs [52] and the FIV Gag chimeras analyzed in the present report indicates that although lentiviral CA proteins share a similar organization (a CA-NTD linked by a flexible region to a CA-CTD together with a conserved MHR), their functional exchange between different lentiviruses is strictly dependent on the context of the recipient Gag precursor.
